# The short‐term postoperative pain and impact upon quality of life of pulpotomy and root canal treatment, in teeth with symptoms of irreversible pulpitis: A randomized controlled clinical trial

**DOI:** 10.1111/iej.14144

**Published:** 2024-09-26

**Authors:** Neha Patel, Iftekhar Khan, Fadi Jarad, Angelo Zavattini, Garrit Koller, Tiago Pimentel, Kazim Mahmood, Francesco Mannocci

**Affiliations:** ^1^ Endodontics Department, Kings College London, Guys Hospital London UK; ^2^ Warwick Medical School University of Warwick Coventry UK; ^3^ School of Dentistry, University of Liverpool Liverpool UK

**Keywords:** endodontic, pulpotomy, root canal treatment

## Abstract

**Background:**

Few studies focus upon patient‐reported outcomes in endodontics.

**Aim:**

To determine whether full pulpotomy offers a less painful, improved health‐related quality of life (HRQoL) compared with root canal treatment (RCT) in cases of irreversible pulpitis (IP) in the 7 days after the treatment.

**Methodology:**

One hundred sixty‐eight participants presenting with symptoms of IP were randomized to either pulpotomy (*n* = 86) or RCT (*n* = 82). Two participants were excluded, 61 participants underwent full pulpotomy with Biodentine (35.7%), 80 had RCT (46.8%), and 25 were randomized to have pulpotomy which progressed to RCT (PRCT) due to uncontrollable bleeding (14.6%). Clinical and radiographic assessments, using CBCT and periapical radiographs, were carried out preoperatively, for the evaluation of the results only CBCT images were used. Pain (VAS) and HRQoL (EQ 5D) assessments were carried out at baseline and Days 1, 3, 5 and 7 post‐baseline. Analysis included descriptive and continuous variables, chi‐squared, Fisher's exact, and two‐sample *t*‐tests.

**Results:**

In pulpotomy and RCT groups, VAS pain decreased significantly over the first week (*p* < .001). The magnitude of reduction was similar in RCT and pulpotomy (*p* = .804), RCT and PRCT (*p* = .179), pulpotomy vs. PRCT (*p* = .144) and in the comparison of combined RCT /PRCT groups (ORCT) with Pulpotomy (0.729). However, the overall level of VAS pain was significantly higher in the PRCT group than in the Pulpotomy (*p* = .045) and RCT group (*p* = .049). Using CBCT, significantly more radiolucencies were found in the PRCT group than in the pulpotomy group and overall teeth presenting with CBCT radiolucencies had significantly higher pain scores (*p* = .015), particularly at Days 1, 3 and 5. There were significant differences in many OHRQoL domains (Questions 1, 6, 11 and 12) between RCT and PRCT groups with higher frequencies of the impact of oral health problems at Day 0 and Day 7 in the PRCT group.

**Conclusion:**

In the treatment of IP, pulpotomy is as effective as RCT in reducing post‐operative pain, and improving QoL and HRQoL, teeth displaying uncontrollable bleeding and periapical radiolucencies detected using CBCT are associated with more intense postoperative pain and lower QoL.

## INTRODUCTION

Patients suffering from irreversible pulpitis are known to experience a high level of pain and discomfort due to the extent of inflammation from the diseased pulp tissue which in turn can impact one quality of life (Cimilli et al., 2012). RCT is the removal of this pulp tissue which allows for tooth retention and pain reduction. The procedure, however, is often perceived negatively by patients and is conceived to be one of the most painful dental treatments (Imura & Zuolo, 1995; Levin et al., 2006). Post‐operative endodontic pain is multifactorial in nature and it is reported that up to 58% of patients experience pain after RCT (Sathorn et al., 2007). This can result in phobias and anxiety becoming a common deterrent to seeking treatment (Watkins et al., 2002) increasing the burden of dental treatment due to ‘problem‐orientated’ visits.

Such treatments tend to be costly and teeth requiring extensive treatment can mean a reduced long‐term prognosis, which not only reduces the patients' confidence (Kvale et al., 2022) but also increases their dental anxiety (Rousseau et al., 2002; van Wijk, 2006).

Recent studies have documented the success of coronal pulpotomies in mature and immature teeth with carious vital pulp exposures, both with signs and symptoms of reversible and irreversible pulpitis (Asgary et al., 2014; Asgary & Eghbal, 2010; Careddu & Duncan, 2021; Duncan, 2022; Eghbal et al., 2020; Taha & Abdulkhader, 2018a, 2018b; Taha et al., 2023) which challenges the decision to perform RCT based on a diagnosis of irreversible pulpitis (Follow‐up of Data from the Swedish Dental Register 2010). In a 2019 systematic review, Cushley et al reported a 95.4% radiographic success and 97.4% clinical success at 1 year following complete pulpotomy in teeth diagnosed with irreversible pulpitis, highlighting the capacity of the pulp tissue to heal and the benefits of maintaining a vital pulp including the preservation of tooth structure, reducing the risk of fracture, reduction in treatment time, the maintenance of a defence system and proprioception (Cushley et al., 2022). It is recognized that a pulpectomy is not the only way of managing irreversible pulpitis and a less invasive treatment could be indicated (AAE, 2021; Duncan et al., 2023), whilst the European Society of Endodontology (ESE) have stated that ‘full pulpotomy be successful in cases where there is partial irreversible pulpitis in the coronal pulp; however better long‐term prospective randomised data are required before this becomes a treatment of choice’ (Duncan et al., 2019).

Research into the extent of post‐operative pain and its impact on quality of life (QoL) is essential in any aspect of clinical practice, particularly when clinicians are faced with multiple treatment options. Oral health‐related quality of life (OHRQoL) is considered to be the most important patient‐reported outcome given it is imperative to overall health‐related quality of life (HRQol) (John, 2020). The use of the oral health impact profile (OHIP‐14) in endodontics has shown that pulpal disease may impact OHRQOL (Liu et al., 2014; Neelakantan et al., 2020) and that orthograde endodontic treatment can improve the quality of life. Many studies have shown that there is a clear benefit for patients undergoing RCT having retained their natural dentition (Vena et al., 2014). Asgary and Eghbal (2013) suggested that pulpotomy could offer patients a less painful alternative to RCT which was also supported by Galani et al. (2017) who found in their randomized controlled clinical trial that pulpotomy with MTA resulted in lower pain score post‐operatively than RCT teeth in cases of carious exposure. There are few studies focused on patient‐reported outcomes, with a majority focused upon clinician‐reported outcomes. Only one other clinical study assessed the HRQoL of patients undergoing VPT and found that on day 1, cases treated with pulpotomy had significantly lower pain levels than after RCT with participants requiring less use of analgesics (Taha et al., 2023).

The objective of this randomized controlled clinical trial was to evaluate whether, in patients suffering from irreversible pulpitis, full pulpotomy offers more pain reduction, and improved QOL and HRQoL compared to RCT in the 7 days after the treatment.

## MATERIALS AND METHODS

As this is a multicentre single‐blinded randomized controlled clinical trial involving clinical intervention on human subjects' ethical approval was obtained from the institutional review board (IRAS 237565) and registered with clinical trials.gov (NCT03956199). The trial was registered on the GSTFT NHS R&D database (PB‐PG‐0817‐20 040). This randomized clinical trial has been written according to Preferred Reporting Items for Randomized Trials in Endodontics (PRIRATE) 2020 guidelines (Nagendrababu et al., 2020).

### Size of patient cohort (based on pain scores)

Power calculations based upon the average post‐operative pain score over time (expressed as an area under the curve) for RCT as reported in Pasqualini et al., 2012, estimated that 156 patients were required (78 per group in a 1:1 randomization) based on clinical efficacy (pain score). The average post‐operative pain score over time (expressed as an area under the curve) for RCT reported in Pasqualini et al. (2012) was 3.2 (from a baseline of 5.6), about 43%. Eren et al. (2018) has reported greater than 70% reductions in pain for pulpotomy compared to RCT, in emergency setting. We therefore considered a 65% reduction (from 3.2 to 1.12) in the pain AUC as being clinically relevant for pulpotomy and estimated a standard deviation (SD) of about 4.5 based on 1000 Monte‐Carlo simulations of the data reported in Pasqualini et al. (2012). Hence, assuming an SD of about 4.5, a mean difference in AUC 1.12 (a 65% reduction) as a result of pulpotomy, at least 80% power and a two‐sided 5% error, we required 70 patients per group (140) in total and 15 patients for the control group. Assuming a further nonresponse rate (for pain diaries) of 10%, an estimated 156 patients are required (78 per group in a 1:1 randomization).

### Recruitment and criteria

The patients were recruited through the acute dental treatment clinic in three hospitals, King's College Dental Hospital, Guy's Hospital and Liverpool Dental Hospital. Potential participants were initially assessed and those with a diagnosis of irreversible pulpitis were further triaged. Patients presenting with symptoms of pain with hot/cold, prolonged (>20 s) or spontaneous pain and tenderness in molar teeth were accepted owing to them fulfilling the inclusion criteria (see Table 1) (Bergenholtz & Spångberg, 2004; Sigurdsson, 2008; Wolters et al., 2017).

**TABLE 1:** Inclusion and exclusion criteria of patients recruited.

Inclusion criteria	Exclusion criteria
1. Patients either male or female over the age of 16 (who can consent for themselves) in good general health	1. The presence of fistulas or swelling
2. Patients with clinical symptoms of irreversible pulpitis who need endodontic treatment	2. Anterior teeth with aesthetic concerns
4. Positive response to thermal stimulation	3. External or internal root resorption
5. Molar teeth	4. Multiple teeth with carious lesions in the same quadrant
	5. Mobile teeth
	6. Pregnant women, in view of requirements for radiographs
	7. Patients younger than 16
	8. Patients unable to give consent
	9. Patients who have been administered antibiotics in the previous month
	10. Immunocompromised patients
	11. Incisor or premolar teeth

Potential participants were approached by the clinician in charge of dental emergency clinics and if they expressed interest in participation, they were approached by the research team who then began the informed consent process. Patient information sheets were given and explained, acknowledging that RCT is considered to be the traditional treatment option and that if the patient is randomized to the pulpotomy group, RCT may still need to be initiated (should bleeding not cease). Volunteers were informed that any further treatment needed would be carried out at the follow‐up visits and they would need return to the hospital after 7 days, 12 and 24 months for clinical and radiographic (PA and CBCT) assessment. Participants were also made aware of the radiation exposure associated with CBCT scans which would be taken preoperatively and at 12 and 24 months to assess the long‐term outcome which will be separately addressed in a follow‐up report. The patients were also informed that, if cuspal coverage was indicated, this would have to be provided by the patients' GDP.

### Consent and data storage

If after discussion and having read the information sheet the patient expressed interest in participation, informed consent was obtained. Patients were allocated a unique identification number, and all data was stored in an encrypted database. The principal investigator maintained a database of the patient's personal data, clinical notes and treatment records. Only the chief investigators at each centre had access to the participants' personal data during the study. Block randomization was performed centrally, at a different site, by the Biostatistics Unit, King's College London Dental Institute, using a minimization algorithm and the techniques coded A and B.

### Assessment and procedure

The study could not be double‐blinded as the dentist needed to identify the technique to apply it and the patient was aware of the difference between the two procedures. The patient age and the presence/absence of lesion (CBCT) were considered as prognostic factors to be balanced during the concealed allocation of patients into each study group. Methods of assessment included clinical evaluation of the pulp status including pulp sensibility tests (electric and thermal tests), palpation and percussion tests, along with the presence of signs of inflammation (pain, abscess, sinus tract and abnormal mobility). Cold tests were carried out with Endo‐Frost (Roeko) sprayed onto a small cotton pledget held by tweezers. Patients were asked to lift their hand once they felt pain and then asked to lower their hand once the pain had completely ceased (duration of pain was recorded by stop watch). Periapical radiographs and cone beam computed tomography (CBCT) were also used to assess the tooth at baseline. All treatment was carried out by 10 postgraduate endodontic students and qualified endodontists working within the hospitals.

### Treatment protocol: Pulpotomy

Caries removal was carried out under local anaesthetic (2% lignospan with adrenaline 1/100 000; Septodont) rubber dam isolation and the aid of dental operating microscopes (three step entree Dental Microscope; Global or 5 step Zumax). In The tooth and rubber dam were wiped down with 5.25% sodium hypochlorite (NaOCl) (Chloraxid). Carious tissue was removed and replacement of the missing walls of the tooth with a composite or glass ionomer restoration was carried out. Before the pulp chamber roof was breached a new sterile bur was used and a full pulpotomy was carried out removing pulp tissue up to the level of the canal orifices with a fast handpiece and diamond but under water irrigation. A cotton pellet soaked in 3% NaOCl was placed into the pulp chamber with a dry pellet on top and left for 2 min until haemostasis was achieved. This was repeated up to five times if bleeding did not cease. After this time if haemostasis was not achieved then RCT was initiated. Biodentine was mixed according to the manufacturer's instructions and placed into the pulp chamber (4 mm thick), after initial setting (12 min) Biodentine was covered with permanent glass–ionomer (Fuji IX glass ionomer cement, GC Corporation) and a layer of composite resin (Herculite Ultra;Kerr Corporation, Ceram X Duo; Dentsply Sirona).

### Treatment protocol: RCT

Local anaesthetic was administered, rubber dam was applied and treatment was carried out with the aid of dental operating microscopes (three‐step entree Dental Microscope; Global or five‐step Zumax). Caries were removed and replacement of the missing walls of the tooth with a composite or glass ionomer restoration was carried out. The pulp chamber was accessed, and irrigation was performed throughout the procedure with 3% NaOCl. Stainless steel K‐Flexofiles (Dentsply Sirona) were used to negotiate the canal to its provisional working length which was determined with an apex locator (Morita Dentaport Root ZX; J Morita) and prepared using nickel‐titanium rotary files (ProTaper Gold, ProTaper Next, Wave One Gold, Dentsply Sirona, Reciproc, Reciproc Blue, VDW). Once the pulp tissue was removed the tooth was dressed with calcium hydroxide paste (using a syringe) (Calcipaste; Cerkamed) followed by PTFE and GIC to close. After 7 days RCT was completed. A penultimate irrigation with 15% or 17% ethylenediaminetetraacetic acid (EDTA) was undertaken followed by a final irrigation with sodium hypochlorite. All canals were filled with gutta‐percha (Dentsply Sirona) using a warm vertical condensation technique and AH Plus Sealer (Dentsply‐Sirona). Composite resin was then used to immediately restore the teeth.

Baseline assessments included:

1. Demographics, including previous history of RCT and/or pulpotomy.
2. Clinical assessments (pain assessment based on pain diary for assessment of symptoms such as pain, tenderness, and swelling).
3. VAS pain scale for assessment of pain.
4. EQ‐5D‐5L (Health‐Related Quality of Life) for assessment of utility and the pain dimension.
5. OHIP‐14 assessment (Oral Health Index Profile) to assess the impact oral health problems have upon an individual's life.
6. Periapical radiographs and CBCT scans.
7. Analgesics taken pre‐operatively.

Assessment and comparison of self‐reported pain and health scores were done using a pain diary (Appendix S1) which the patient was instructed to complete at five different time points: Pre‐operatively before treatment was carried out (done in the clinic together with the operating dentist), day 1, day 3, day 5 and day 7 (see Table 2). The operating dentists were trained in the use of the pain diary by one of the investigators. Patients completed the preoperative portion (first time point) of the pain diary in the presence of the operating dentist to ensure that they understood how to complete both the EQ‐5D questionnaire as well as the VAS instrument. The pain diary consisted of an OHIP‐14, and EQ‐5D‐5L questionnaire and a visual analogue scale (VAS) for each time point (Appendix S1; see Table 2).

**TABLE 2:** Assessments undertaken at specific time points.

Baseline (pre‐op)	Day 1	Day 3	Day 5	Day 7
Demographics	EQ‐5D‐5L	EQ‐5D‐5L	EQ‐5D‐5L	Clinical assessment
Clinical assessment	VAS	VAS	VAS	EQ‐5D‐5L
EQ‐5D‐5L	Pain medication	Pain medication	Pain medication	VAS
OHIP‐14				OHIP‐14
VAS				Pain medication
Pain medication				
PA				
CBCT				

### Oral health‐related quality of life assessment

The Oral Health Impact Profile (OHIP‐14) aims to capture seven dimensions related to oral health (see Table 3): functional limitation, physical pain, psychological discomfort, physical disability, psychological disability, social desirability and handicap. These concepts encompass outcomes that impact and can disturb people's lives.

**TABLE 3:** Dimensions and quality of life (QoL) items comprising the OHIP‐14 instrument.

Dimension	Question	Weight
Functional limitation	1. Have you had trouble pronouncing any words because of problems with your teeth, mouth or dentures?	0.51
	2. Have you felt that your sense of taste has worsened because of problems with your teeth, mouth of dentures?	0.49
Physical pain	3. Have you had painful aching in your mouth?	0.34
	4. Have you found it uncomfortable to eat any foods because of problems with your teeth, mouth or dentures?	0.66
Psychological discomfort	5. Have you been self‐conscious because of your teeth, mouth or dentures?	0.45
	6. Have you felt tense because of problems with your teeth, mouth or dentures?	0.55
Physical disability	7. Has your diet been unsatisfactory because of problems with your teeth, mouth or dentures?	0.52
	8. Have you had to interrupt meals because of problems with your teeth, mouth or dentures?	0.48
Psychological disability	9. Have you found It difficult to relax because of problems with your teeth, mouth or dentures?	0.6
	10. Have you been a bit embarrassed by problems with your teeth, mouth or dentures?	0.4
Social disability	11. Have you been a bit irritable with other people because of problems with your teeth, mouth or dentures?	0.62
	12. Have you had difficulty doing your usual jobs because of problems with your teeth, mouth or dentures?	0.38
Handicap	13. Have you felt that life in general was less satisfying because of problems with your teeth, mouth or dentures?	0.59
	14. Have you been totally unable to function because of problems with your teeth, mouth or dentures?	0.41

*Note*: Responses are made on a 5‐point scale, coded 0 = never, 1 = hardly ever, 2 = occasionally, 3 = fairly often, 4 = very often. Within each dimension, coded responses can be multiplied by weights to yield a subscale score.

### Quality of life assessment

HRQoL was assessed using the EuroQoL‐developed EQ‐5D‐5L (Appendix S1). The instrument has five dimensions—mobility, self‐care, usual activities, pain/discomfort and anxiety/depression. Each dimension has five levels—no problems, slight problems, some problems, moderate problems and extreme problems. This was assessed at baseline, days 1, 3, 5 and 7.

### Pain assessment

Patients were asked to record their pain on a 100 mm long VAS scale which spans from 0 (no pain) to 100 (worst pain) by marking a cross at each required time point. Scores were measured with a ruler (see Appendix S1).

### Statistical analysis

A descriptive analysis of categorical variables (absolute and relative frequencies) and continuous (mean, standard deviation, range, median and quartiles) overall and by treatment group were generated. For categorical responses, chi‐square and Fisher's exact test was used to compare treatment groups. For continuous outcomes, a General Linear Model (GLM) was used to estimate mean differences between groups along with 95% confidence intervals. Where departures from normality occurred, non‐parametric methods were used. All statistical tests were provided at the two‐sided 5% level without adjustment for multiplicity, unless stated otherwise.

## RESULTS

**FIGURE 1:**
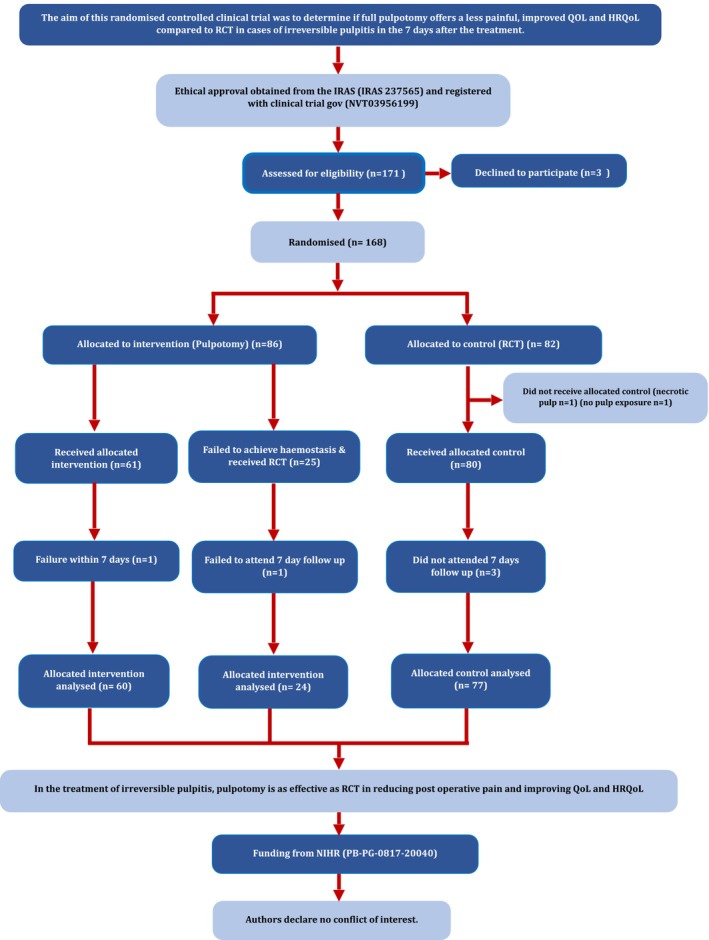
Experimental flowchart.

A total of 171 participants were assessed for eligibility of which three declined to participate and 168 were recruited and randomized to participate in this study between September 2019 and July 2022 (see Figure 1). Sixty‐one participants underwent pulpotomy (35.7%), 80 had RCT (46.8%) and 25 were randomized to have pulpotomy which progressed to RCT (PRCT) due to uncontrollable bleeding (14.6%). A fourth group (ORCT) was included in the analysis consisting of the combined RCT groups (RCT and PRCT), the four groups were found to be homogenous in terms of demographic and clinical variables. One participant was withdrawn from the RCT group due to no pulp exposure which led to the successful attempt to preserve the vitality of the tooth with caries removal only and another as the tooth was found to be necrotic. A total of 84 males (49.1%) and 87 females (50.9%), with an age range between 18 and 75 years, were included in the trial. The mean age was 37 years and the median was 34.8 years. 19.2% of teeth treated were upper first molars, 18% were upper second molars, 42.5% lower first molars and 20.4% were lower second molar teeth. In 23 teeth (12.8%) one wall was missing, in 105 teeth (64%) two were missing and in 38 (23.2%) three or more walls were missing. The average time for the procedure (which included the placement of a plastic restoration) was 73.8 min in the pulpotomy group and 100.3 in the RCT group (Table 4).

**TABLE 4:** Distribution of teeth across categories.

	Total (*N* = 171)	Pulpotomy (*N* = 61)	RCT (*N* = 80)	PRCT (*N* = 25)	*p* Values (chi‐square)
*Gender*					
Male	84 (49.1%)	27 (44.3%)	41 (51.3%)	12 (48%)	.713
Female	87 (50.9%)	34 (55.7%)	39 (48.8%)	13 (52%)	
*Position*					
Total	167 (100%)	61 (100%)	79 (100%)	24 (100%)	.575
Upper 6	32 (19.2%)	14 (23%)	16 (20.3%)	2 (8.3%)	
Upper 7	30 (18%)	8 (13.1%)	16 (20.3%)	4 (16.7%)	
Lower 6	71 (42.5%)	29 (47.5%)	30 (38%)	12 (50%)	
Lower 7	34 (20.4%)	10 (16.4%)	17 (21.5%)	6 (25%)	
*Walls involved*					
Total	164 (100%)	61 (100%)	78 (100%)	25 (100%)	.029
1 Wall	21 (12.8%)	7 (11.5%)	7 (9%)	7 (28%)	
2 Walls	105 (64%)	38 (62.3%)	57 (73.1%)	10 (40%)	
3 + Walls	38 (23.2%)	16 (26.2%)	14 (17.9%)	8 (32%)	
AV. time for procedure (first appointment)					
Mean (min)	91.2	73.8	100.3	104	<.001
SD	34.0	20.4	34.9	41.3	
*CBCT status*					
Normal	72 (43.6%)	34 (55.7%)	32 (41%)	5 (20.8%)	.048
Abnormal (slight enlargement)	55 (33.3%)	17 (27.9%)	28 (35.9%)	10 (41.7%)	
Abnormal (Lesion)	38 (23%)	10 (16.4%)	18 (23.1%)	9 (37.5%)	

### Primary outcome

#### Pain assessment: Visual Analogue Scale

##### Pulpotomy versus RCT

**FIGURE 2:**
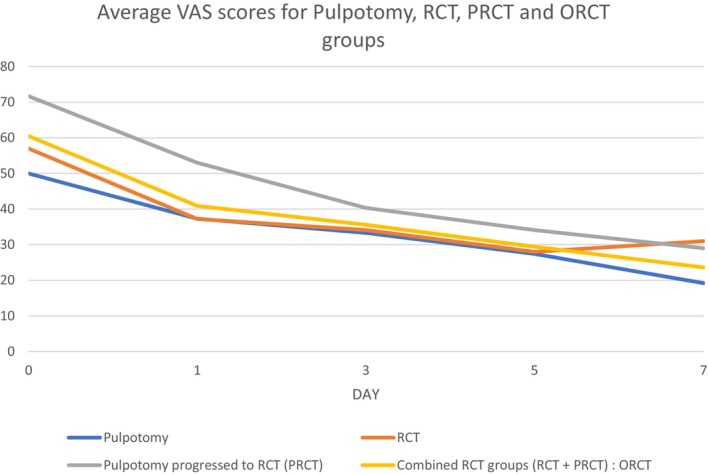
Line graph showing reduction in mean pain Visual Analogue Scale (VAS) scores over 7 days across the four groups.

Mean pain scores (AUC) for pulpotomy versus RCT were 221.55 versus 243.9 (see Table 5). In both the RCT and pulpotomy group, pain decreased significantly over the 1st week after the procedure (*p* < .001; Figure 2). The magnitude of the reduction was similar in the comparison of RCT and pulpotomy groups (*p* = .804). No differences were found between either pulpotomy or RCT group in any day (see Tables 6 and 7).

**TABLE 5:** Mean pain Visual Analogue Scale (VAS) over time by group and area under the curve (AUC).

	Pulpotomy	RCT	PRCT	ORCT
	Mean VAS scores			
Day 0	50	57.0	71.7	60.5
Day 1	37.3	37.2	53	40.9
Day 3	33.3	34.1	40.3	35.6
Day 5	27.4	28	34.1	29.4
Day 7	19.2	31	29	23.6
Area under curve	221.55	243.9	293.15	245.2

*Note*: ORCT [OVERALL RCT – RCT (Root canal treatment) + PRCT (pulpotomy progressed to root canal treatment)].

Abbreviation: PRCT, pulpotomy progressed to RCT.

**TABLE 6:** anova model testing of mean pain Visual Analogue Scale (VAS) scores between groups.

	Pulpotomy	RCT	PRCT	ORCT/pulpotomy
	Comparison of three groups			Comparison of two groups
Effect time	*p* < .001***			*p* < .001***
Effect group	*p* = .088			*p* = .221
Effect time × Group	*p* = .394			*p* = .729

*** *p* < .001.

**TABLE 7:** Pain Visual Analogue Scale (VAS) over time by group: results from multiple comparisons with Bonferroni's correction.

	RCT versus pulpotomy	ORCT versus pulpotomy	PRCT versus pulpotomy	RCT versus PRCT
	*p*‐Value	*p*‐Value	*p*‐Value	*p*‐Value
Day 0	.131	**.023** *	**.004** **	**.039** *
Day 1	.995	.465	**.045** *	**.022** *
Day 3	.864	.636	.356	.369
Day 5	.892	.660	.356	.369
Day 7	.626	.368	.145	.237

* *p* < .05.

** *p* < .01.

Bold values indicate statisical signifiacnce has been reached (*p* < .05)

##### RCT versus PRCT

Mean pain scores (AUC) for RCT versus PRCT were 243.9 versus 293.15 (see Table 5). The magnitude of the reduction was similar (*p* = .179) and significant differences were noted at days 0 (*p* = .039) and 1 (*p* = .022; see Table 7). The overall level of VAS pain was significantly higher in the PRCT group than in the RCT (*p* = .049).

##### ORCT versus pulpotomy

Mean pain scores (AUC) for ORCT versus pulpotomy were 245.2 versus 221.55 (see Table 5). Pain reduction over the first 7 days was similar between groups (*p* = .729) with no other differences noted (Figure 2 and see Table 6).

##### Pulpotomy versus PRCT

Mean pain scores (AUC) for pulpotomy versus PRCT were 221.55 versus 293.15 (Table 5). Reduction in pain was similar between groups (*p* = .144; Figure 2). Significant differences between pulpotomy and PRCT at days 0 (*p* = .004) and 1 (*p* = .0454) were found (see Table 7). The overall level of VAS pain was significantly higher in the PRCT group than in the pulpotomy group (*p* = .045).

### Secondary outcomes

#### Health‐Related Quality of Life Assessment (HRQoL): EQ‐5D‐5D

Overall, the average mean EQ‐5D‐5L scores reduced in each group over the 7 days (Table 8). The EQ5 health score increased significantly over the 1st week after the procedures (*p* < .001), the magnitude of improvement was similar when comparing all groups with no overall differences between any group at any day (see Table 9). Problems with usual activities reduced significantly when comparing all groups and to similar magnitudes, however, severity of problems with usual activities was significantly higher in the RCT group than pulpotomy (*p* = .023) and in the ORCT group than pulpotomy group (*p* = .008) as overall averages (see Table 10). Pain and discomfort significantly improved when comparing all groups, however, there was a significantly higher level of pain overall in the PRCT group compared to the pulpotomy group (*p* = .048). Again, problems with anxiety and depression reduced significantly in all groups.

**TABLE 8:** EQ‐5D‐5L average values of the five dimensions over time intervals by group.

	Total	Pulpotomy	RCT	PRCT	ORCT
*Day 0*					
Mean	1.71	1.62	1.69	1.99	1.76
SD	0.50	0.49	0.42	0.70	0.51
Median	1.60	1.60	1.60	1.80	1.70
*Day 1*					
Mean	1.49	1.41	1.52	1.60	1.54
SD	0.47	0.39	0.50	0.51	0.50
Median	1.40	1.40	1.40	1.40	1.40
*Day 3*					
Mean	1.43	1.34	1.46	1.53	1.48
SD	0.46	0.34	0.47	0.65	0.51
Median	1.20	1.20	1.20	1.20	1.20
*Day 5*					
Mean	1.33	1.27	1.35	1.41	1.37
SD	0.40	0.31	0.41	0.56	0.45
Median	1.20	1.20	1.20	1.20	1.20
*Day 7*					
Mean	1.25	1.20	1.25	1.34	1.27
SD	0.37	0.25	0.37	0.58	0.43
Median	1.20	1.20	1.10	1.20	1.20

**TABLE 9:** EQ‐5D‐5L Health score over time by group: Results from multiple comparisons with Bonferroni's correction.

	RCT/pulpotomy	ORCT/pulpotomy	PRCT/pulpotomy	RCT/PRCT
	*p*‐Value	*p*‐Value	*p*‐Value	*p*‐Value
Day 0	.870	.472	.112	.086
Day 1	.355	.233	.230	.472
Day 3	.407	.447	.836	.680
Day 5	.240	.255	.636	.699
Day 7	.602	.631	.892	.798

* *p* < .05.

** *p* < .01.

*** *p* < .001.

Bold values indicate statisical signifiacnce has been reached (*p* < .05)

**TABLE 10:** EQ‐5D‐5L dimensions over time by group: results from Bruner‐Langer model for main effects and interaction.

	RCT/pulpotomy	ORCT/pulpotomy	PRCT/pulpotomy	RCT/PRCT
	*p*‐Value	*p*‐Value	*p*‐Value	*p*‐Value
*Mobility*				
Time	**.011** *	.131	.441	.317
Group	.661	.841	.768	.579
Time × group	.464	.716	.324	.157
*Self‐care*				
Time	.170	.258	.722	.172
Group	.640	.509	.527	.688
Time × group	.358	.490	.720	.172
*Usual activites*				
Time	**<.001** ***	**<.001** ***	**<.001** ***	**<.001** ***
Group	**.023** *	**.008** **	.056	.687
Time × group	.358	.249	.161	.185
*Pain/Discomfort*				
Time	**<.001** ***	**<.001** ***	**<.001** ***	**<.001** ***
Group	.349	.135	**.048** *	.146
Time × group	.853	.622	.178	.260
*Anxiety/Depression*				
Time	**<.001** ***	**<.001** ***	**<.001** ***	**<.001** ***
Group	.101	.076	.344	.949
Time × group	.618	.375	.408	.569

* *p* < .05.

** *p* < .01.

*** *p* < .001.

#### Assessment of impact oral health problems have upon an individual's life (OHRQoL): OHIP‐14

Most impacts of oral health problems improved significantly over 7 days (*p*‐value for time <.05). Only questions #1 and #2 remained stable. Most of these significant changes were similar in all groups (*p*‐value for interaction >.05).

OHIP‐14 scores improved significantly over the 1st week after the procedures (*p* < .001) and the magnitude was similar in RCT and pulpotomy groups (*p* = .668), PRCT and RCT (*p* = .126), pulpotomy and RCT groups (*p* = .154), ORCT and pulpotomy groups (*p* = .901).

There were significant differences in some OHRQoL domains (questions 1, 6, 11 and 12) between RCT and PRCT groups with higher frequencies of impact of oral health problems at day 0 and day 7 in the PRCT group. This was also found when comparing pulpotomy and ORCT group (questions 1, 2, 6, 11, and 14) showing ORCT patient group to experience a more negative impact on quality of life.

#### Other comparisons

##### Length of procedure

Overall, longer treatment times were observed in the RCT, PRCT and ORCT groups compared to pulpotomy (*p* < .001). Differences were also found in overall OHIP‐14 scores, with more significant improvements associated with a longer treatment time (over 100 min) in the RCT group (*p* = .029) and in the ORCT group (*p* = .013).

##### Presence of pre‐operative radiolucency in CBCT

A periapical lesion was defined as a radiolucency associated with the radiographic apex of the root, which was at least twice the width of the periodontal ligament space (Bornstein et al., 2011; Low et al., 2008). 43.6% of cases presented with no periapical changes, 33.3% with slight widening of the PDL and 23% with an established periapical lesion.

Overall, there were significantly more radiolucencies in the PRCT group compared to the pulpotomy group. Preoperative status (normal, widening of the lamina dura and radiolucency) comparison revealed that overall teeth presenting with radiolucencies had significantly higher pain scores (*p* = .015), particularly at days 1, 3 and 5.

Pain reduction in teeth presenting with a radiolucency in the pulpotomy group occurred later (post‐operative day 3) compared to normal and abnormal preoperative status). EQ‐5D‐5L scores revealed that those presenting with radiolucencies had significantly more problems with usual activities over the 7 days (*p* = .035) in the RCT group. Pain (VAS scores) was significantly higher in the RCT group when a lesion was present (*p* = .002), and varying patterns were noted over the 7 days in usual activities (EQ‐5D‐5L) across groups with more problems in those presenting with radiolucencies (*p* = .035).

##### Pre‐operative pain medication

A total of 55 patients reported the use of pain medication preoperatively. The effect of each type of painkiller was analysed in the total sample due to the low sample size per treatment group. No significant differences were noted in those patients' taking paracetamol or other pain medications. In those who took ibuprofen, the EQ‐5D score increased significantly over the 7 days after the procedure (*p* = .002) and the magnitude of increment was different in those who did and did not take ibuprofen (*p* = .004).

##### Pain medication

In the pulpotomy group, 63.3% of patients took medication, 73.9% in the PRCT group and 57.7% in the RCT group with no significant differences noted.

##### Patient race

In the pulpotomy group, when comparing White and BME patients, the VAS pain decreased significantly over the first week (*p* < .001), but the magnitude of reduction was higher in the BME group in the first 5 days (*p* = .002).

##### Bleeding time

Within the pulpotomy group, no difference in pain reduction, OHIP 14 and EQ5‐5D‐5L, was found between patients with bleeding time above or below 5 min.

## DISCUSSION

This multicentre randomized controlled clinical trial showed that pulpotomy is an effective treatment for short‐term pain relief in cases of irreversible pulpitis, with a comparable pain reduction at day 1, 3, 5 and 7 for RCT and pulpotomy, the pain reduction at longer recalls will be presented in a future clinical and radiographic outcome study paper.

Patients randomized to pulpotomy who had pulpal bleeding for more than 10 min upon access and were therefore re‐assigned to the RCT group, experienced higher levels of pain pre‐operatively and post‐operatively in the first 24 h compared to patients in the other groups. Teeth within this group also presented with a higher number of preoperative radiolucencies indicating that excessive bleeding and lesions tend to present together and are associated with more post‐operative pain in the short term. This is of particular significance considering that, to the authors' knowledge, this is the first pulpotomy study in which a pre‐operative radiographic assessment was undertaken using CBCT. In this study, we did not consider periapical radiolucencies as an exclusion criterion given reports have shown complete resolution of periapical radiolucencies after pulpotomy (Asgary et al., 2016; Foreman, 1980; Jiang et al., 2016; Moule & Oswald, 1983). Apical periodontitis develops soon after pulp necrosis, however, portions of the radicular pulp can remain vital even when a radiolucency is detected radiographically (Lin et al., 1984; Ricucci et al., 2013) suggesting that pulpotomy can produce favourable outcomes and may serve as a viable alternative to RCT in teeth affected by apical periodontitis (Duncan et al., 2023).

Similarly, the QoL questionnaire showed a more negative impact of this condition on the patient's life. To the authors' knowledge this is the first study which describes the pain and impact of QoL of severe pulpal inflammation associated with prolonged bleeding.

Cases with prolonged bleeding could have been excluded from the trial however, the statistical analysis was undertaken using an MMRM (mixed model of repeated measures) approach rather than excluding the cases with prolonged bleeding, which would bias the results as it would be selective and make the experiment even less randomized by reporting data from operators who may have been pre‐disposed to report better results. In cases of prolonged bleeding, it is advisable to prescribe stronger pain medications and inform patients of the higher level of pain and lower QoL they are likely to continue to experience postoperatively, particularly in the first 24 h after the treatment. Pre‐ and postoperative pain has also been associated with worse treatment outcomes (Law et al., 2014; Pak & White, 2011), this potential association will be tested in the outcome part of this investigation.

In terms of pain relief, the results of the present study are in agreement with those of Eghbal et al. (2020), as no statistically significant difference was found between pulpotomy and RCT. In contrast, Asgary and Eghbal (2010) and Taha et al. (2023) found that pulpotomy provided a better pain‐reducing effect. Several preoperative factors which were found to be homogeneously distributed in our study (including ethnicity, qualification, employment status, smoking, anxiety levels, diabetes and other medical conditions) were not tested in previous studies. The potential lack of homogeneity of the two groups in previous studies might be one of the causes of this difference, in this respect, it is not surprising that the largest study available (Eghbal et al., 2020) supports our conclusions, as the high number of patients recruited may be associated with a more homogenous distribution of uncontrolled factors.

In a recent meta‐analysis, Tomson et al. (2022) found that on the 7th day postoperatively, patients had similar pain levels irrespective of the treatment intervention, reflecting the results of this randomized controlled trial.

The reduction in pain after pulpotomy has been attributed to the ‘reduction in local tissue pressure, inflammatory mediator concentrations and the severing of terminal endings of nociceptive sensory neurons’ (Rosenberg, 2002). However, this is also achieved after RCT, which may account for a similar reduction in pain.

The HRQoL assessment showed significant improvements over time in all groups, with significant differences in favour of pulpotomy concerning the patient's ability to resume usual activities post‐treatment. In relation to the effect of RCT and pulpotomy on the patients' quality of life EQ‐5D5L measures were used. One major feature of the EQ‐5D tool is that the health states obtained from the questionnaire may be converted into a single index value with country‐specific value sets. This facilitates calculating and comparing quality‐adjusted life years (QALYs) between pulpotomy, primary RCT and potential extraction. The QALY is a measure of disease burden including the quality and quantity of life lived, with one QALY equating to 1 year in perfect health. This can then be used for economic evaluations of healthcare interventions and possibly aid in allocating healthcare resources more effectively and efficiently.

When carrying out treatment, there was a large proportion of teeth randomized to the pulpotomy group which failed to achieve haemostasis, suggesting that inflammation was extending apically to the coronal portion of the pulp, the high number of cases included in the PRCT group suggests that a diagnosis of irreversible pulpitis is likely to have a limited time frame before the inflammatory process spreads to the radicular portion of the pulp. Any delay between the patient presentation and the treatment being performed has a potential impact on whether a coronal pulpotomy can be performed, and the intraoperative assessment of the pulp tissue remains a valuable indicator of the ideal course of treatment. However, the uncertainty of our present pulp testing methods to determine the actual inflammatory condition of the pulp leads to a lack of a clear treatment plan (whether pulpotomy can be performed or not) which is likely to create some anxiety amongst patients. Pre‐operatively there is no definitive tool that can determine how significantly the inflammation has spread into the pulp, however, the advancement in the detection of biological inflammatory markers might help identify the extent of inflammation and determine the success of treatment (Rechenberg et al., 2016).

Most pulpotomy studies (Asgary & Eghbal, 2010; Taha & Abdelkhader, 2018a; Taha et al., 2023) do not report the outcome of recruited patients which were later excluded due to excessive bleeding. In Taha & Abdelkhader (2018b) study, only 13% of cases could not achieve haemostasis which contrasts with the present study, whereby one in four patients randomized to pulpotomy had to be re‐allocated to the RCT group due to prolonged bleeding. This indicates that full pulpotomy is not suitable in approximately 25% of the patients presenting with symptoms of irreversible pulpitis; to prevent the increased loss of tooth structure associated with an RCT, and considering that CBCT‐based clinical trials (Ali et al., 2020) show that almost 50% of the teeth presenting with symptoms of reversible pulpitis treated with caries excavation techniques require some form of pulpal intervention within 2 years, it is perhaps advisable to consider the timely use of pulp vitality preservation procedures such as partial if possible (Careddu & Duncan, 2021)or full pulpotomy when the intensity of the patients' symptoms has not yet reached the level which is indicative of irreversible pulpitis: in fact, the 3‐year success rate of pulpotomy was found to be in the region of 85% (Cushley et al., 2022).

Only one other paper has looked at the differences in post‐operative pain and QoL after pulpotomy and RCT in cases of irreversible pulpitis (Taha et al., 2023) highlighting the knowledge gap. Cushley et al., 2022 recognized that though some studies have used unspecified questionnaires, the majority of studies' pain reports were from patient histories, without the use of a measurement instrument, demonstrating a lack of standardization in pain reporting. Outcomes related to quality of life were also, again reported from patient histories with no studies having adopted global quality of life measures (Cushley et al., 2022). Pain perception is a highly individualized and multifactorial phenomenon, and pain reporting is influenced by factors beyond the experimental procedures. Pain assessment is also prone to errors and challenges relying on the assumption that pain intensity is a continuous variable. In this study, RCT was completed over two visits to reflect the realistic treatment protocols in clinical practice as patients suffering from irreversible pulpitis are more likely to present in an emergency slot where there are time constraints and rarely the opportunity to carry out RCT in one visit. Pulpotomy was demonstrated to offer a shorter treatment duration compared to RCT. It is also noteworthy that patients in the RCT group required a second treatment session, highlighting the advantages of pulpotomy not only in terms of time management but also in enhancing patient comfort. The assessment of pain both pre and post‐operatively was self‐reported by participants thereby, reducing any bias (McCahon et al., 2005) however, it is important to acknowledge the possibility of the Hawthorne effect. Participants awareness of their pain HRQoL and QoL being monitored may have altered their reporting to that which they believed the researchers wished to see or comply with their perceived study goals. To mitigate this, patients were not informed of the treatment that had been performed, however, they may have become aware during the procedure particularly if they had previous experience of RCT.

A visual analogue scale (VAS) was used to measure the intraoperative pain felt during RCT, which allows the patient to give an overall rating of their pain. This method has been proven to work well for assessing pain, including dental pain, in clinical settings (Kayaoglu et al., 2016; Yucel et al., 2018) and is a valid and reliable method that is widely used in the endodontic literature (Hargreaves & Keiser, 2002; Martin‐Gonzalez et al., 2012; Sathorn et al., 2008; Segura‐Egea et al., 2009). The VAS scale has been shown to be a highly reliable tool in the assessment of acute pain in adults (Bijur et al., 2001). Garra et al., 2010 also showed that the VAS scale was more sensitive to changes in pain and a more informative method in comparison to other scales. The use of OHRQoL in research can vary according to what specifically is being assessed. In this case, the oral health impact profile (OHIP) is targeted toward oral health conditions in comparison to the Short Form Health Survey (SF‐36) which is a more generic QoL measure (Lee et al., 2007) and might not be as sensitive to the subtle differences between health conditions. The OHRQOL is also the most used and well‐established method to assess the changes to QoL that occur after endodontic interventions (Liu et al., 2014; Montero et al., 2015). Health scores in this study were obtained using the EQ‐5D questionnaire which is a standardized measure of health status developed by the EuroQoL Group to provide patient‐reported outcomes (PRO) and a simple generic measure of health for clinical and economic appraisal The EQ‐5D is the most popular generic PRO questionnaire in the world (Devlin & Brooks, 2017). It is well documented that the EQ‐5D provides and reliable and valid measure in several areas (Janssen et al., 2011; Pickard et al., 2007).

The similarity in the levels of pain post‐operatively does show that amputation of the pulp tissue can effectively manage pain (Hasselgren & Reit, 1989) rather than total removal whereby haemostasis can be achieved in the coronal portion. Such outcomes in cases of symptomatic irreversible pulpitis can be ascribed to the fact that they can modulate immune responses and lower the amounts of cytokines that cause inflammation in the dental pulp (Chang et al., 2014) and that inflammation in histologic studies is confined to the coronal portion (Ricucci et al., 2014). Additionally, new‐generation tricalcium silicate materials have anti‐inflammatory properties that help to reverse the remaining inflammation and preserve a healthy pulp tissue afterwards (Meschi et al., 2019). Post‐operative pain research has shown that the presence of apical periodontitis or widening of the PDL is associated with more post operative pain (Eghbal et al., 2020), irrespective of whether the patient underwent pulpotomy or RCT which has been attributed to the higher levels of inflammation intrapulpally and periradicularly (Zhang et al., 2016) as reflected in this study.

## CONCLUSION

This study shows that in managing cases with irreversible pulpitis, pulpotomy is as effective as RCT in reducing post‐operative pain and improving QoL and HRQoL. It also shows that in cases of irreversible pulpitis excessive bleeding and periapical radiolucencies tend to present together and are associated with more post‐operative pain in the short term. In such cases of prolonged bleeding, it is advisable to prescribe stronger pain medications and inform patients of the higher level of pain and lower QoL they are likely to continue to experience postoperatively, particularly in the first 24 h after the treatment.

## AUTHOR CONTRIBUTIONS

Neha Patel: Investigation, Writing—Original Draft, Data Curation. Iftekhar Khan: Conceptualization, Methodology, Formal analysis. Fadi Jarad: Conceptualization, Methodology. Angelo Zavattini: Data Curation. Garrit Koller: Data Curation. Tiago Pimentel: Methodology, Data Curation. Kazim Mahmood: Data Curation. Francesco Mannocci: Conceptualization, Methodology, Writing—Review and Editing, Supervision.

## FUNDING INFORMATION

Funding for this study was supplied by the NIHR (PB‐PG‐0817‐20 040).

## CONFLICT OF INTEREST STATEMENT

All authors declares that there is no conflict of interest in relation with this research.

## ETHICS STATEMENT

Ethical approval was obtained from the institutional review board (IRAS 237565) and registered with clinicaltrials.gov (NCT03956199). The trial was registered on the GSTFT NHS R&D database (PB‐PG‐0817‐20 040).

## REPORTING GUIDELINES

This randomized clinical trial has been written according to Preferred Reporting Items for Randomized Trials in Endodontics (PRIRATE) 2020 guidelines (Nagendrababu et al., 2020). See attached flow chart and checklist.

## Supporting information


Appendix S1.

## Data Availability

The data that support the findings of this study are available from the corresponding author, [NP], upon reasonable request.
