# Supplementary material for: The short‐term postoperative pain and impact upon quality of life of pulpotomy and root canal treatment, in teeth with symptoms of irreversible pulpitis: A randomized controlled clinical trial
Source: Int Endod J. 2024 Sep 26;58(1):55–70. doi: 10.1111/iej.14144 (PMC11629050; doi:10.1111/iej.14144)
Supplement: Supplementary file 1 — Appendix S1. [file IEJ-58-55-s001.docx]

APPENDIX 1. Pain Diary


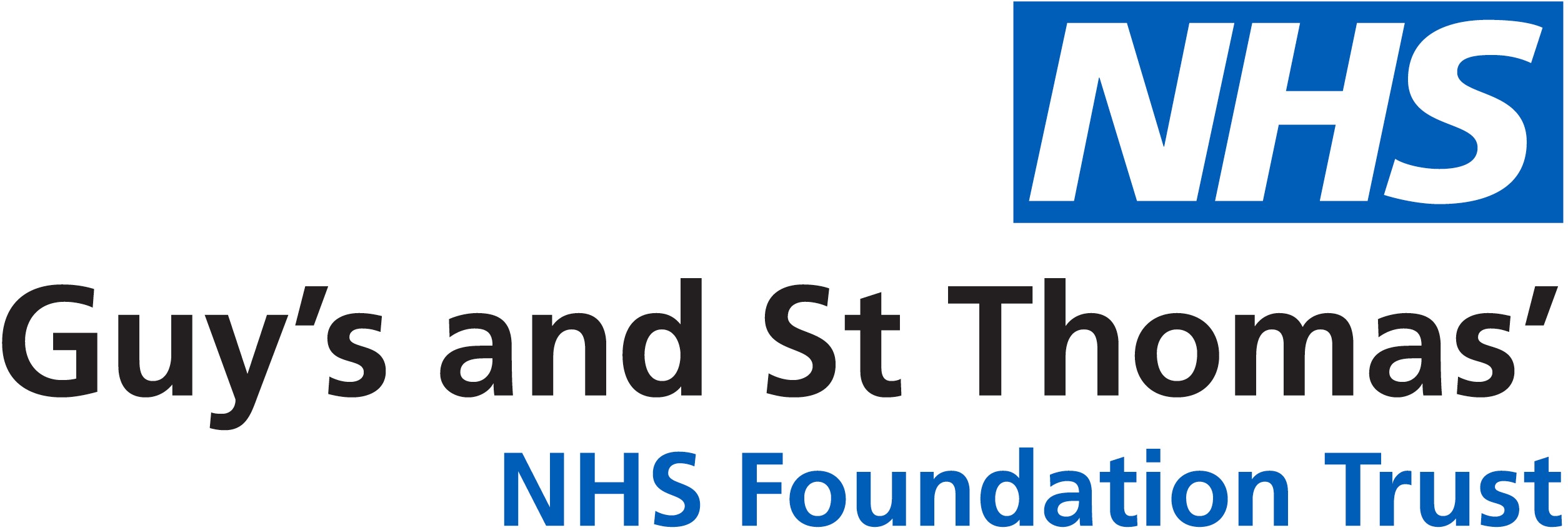


PAIN DIARY

**Attach patient label**

Patient’s Study ID

## Floor 25, Tower Wing

Guy’s Hospital, Great Maze Pond Road London SE1 9RT


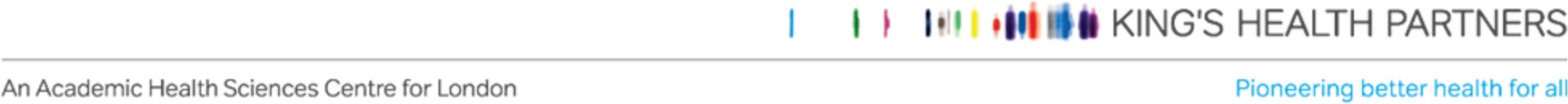


**PLEASE NOTE: In completing this questionnaire, please be assured that all information which would permit identification of individual patients will be held in strict confidence and will be used only by persons engaged in and for the purposes of the study. Information will not be disclosed or released to others for any other purpose. The information collected will be used only to prepare statistical summaries.**

**If a question is not applicable, please write in ‘not applicable’ and if you have any questions about a particular item, you may discuss them with a member of the team during the review visit.**

***Oral Health-Related Quality of Life (OHIP – 14)***

|  | **Never** | **Occasionally** | **Fairly often** | **Very often** |
| --- | --- | --- | --- | --- |
| **1. Have you had trouble pronouncing any words because of problems with your teeth, mouth or dentures?** |  |  |  |  |
| **2. Have you felt that your sense of taste has worsened because of problems with your teeth, mouth or dentures?** |  |  |  |  |
| **3. Have you had painful aching in your mouth?** |  |  |  |  |
| **4. Have you found it uncomfortable to eat any foods because of problems with your teeth, mouth or dentures?** |  |  |  |  |
| **5. Have you been self conscious because of your teeth, mouth or dentures?** |  |  |  |  |
| **6. Have you felt tense because of problems with your teeth, mouth or dentures?** |  |  |  |  |
| **7. Has your diet been unsatisfactory because of problems with your teeth, mouth or dentures?** |  |  |  |  |
| **8. Have you had to interrupt meals because of problems with your teeth, mouth or dentures?** |  |  |  |  |
| **9. Have you found it difficult to relax because of problems with your teeth, mouth or dentures?** |  |  |  |  |
| **10. Have you been a bit embarrassed because of problems with your teeth, mouth or dentures?** |  |  |  |  |
| **11. Have you been a bit irritable with other people because of problems with your teeth, mouth or dentures?** |  |  |  |  |

| **12. Have you had difficulty doing your usual jobs because of problems with your teeth, mouth or dentures?** |  |  |  |  |
| --- | --- | --- | --- | --- |
| **13. Have you felt that life in general was less satisfying because of problems with your teeth, mouth or dentures?** |  |  |  |  |
| **14. Have you been totally unable to function because of problems with your teeth, mouth or dentures?** |  |  |  |  |

**PRE-OP DATE & TIME:**

Under each heading, please tick the ONE box that best describes your health TODAY.

# MOBILITY

I have no problems in walking about ❑

I have slight problems in walking about ❑

I have moderate problems in walking about ❑

I have severe problems in walking about ❑

I am unable to walk about ❑

# SELF-CARE

I have no problems washing or dressing myself ❑

I have slight problems washing or dressing myself ❑

I have moderate problems washing or dressing myself ❑

I have severe problems washing or dressing myself ❑

I am unable to wash or dress myself ❑ **USUAL ACTIVITIES** *(e.g. work, study, housework, family or leisure activities)*

I have no problems doing my usual activities ❑

I have slight problems doing my usual activities ❑

I have moderate problems doing my usual activities ❑

I have severe problems doing my usual activities ❑

I am unable to do my usual activities ❑

# PAIN / DISCOMFORT

I have no pain or discomfort ❑

I have slight pain or discomfort ❑

I have moderate pain or discomfort ❑

I have severe pain or discomfort ❑

I have extreme pain or discomfort ❑

# ANXIETY / DEPRESSION

I am not anxious or depressed ❑

I am slightly anxious or depressed ❑

I am moderately anxious or depressed ❑

I am severely anxious or depressed ❑

I am extremely anxious or depressed ❑

*UK (English) © 2009 EuroQol Group EQ-5D™ is a trade mark of the EuroQol Group*

**PRE-OP DATE & TIME:**

Under each heading, please tick the ONE box that best describes your health TODAY.

# MOBILITY

I have no problems in walking about ❑

I have some problems in walking about ❑

I am confined to bed ❑

# SELF-CARE

I have no problems washing or dressing myself ❑

I have some problems washing or dressing myself ❑

I am unable to wash or dress myself ❑ **USUAL ACTIVITIES** *(e.g. work, study, housework, family or leisure activities)*

I have no problems doing my usual activities ❑

I have some problems doing my usual activities ❑

I am unable to perform my usual activities ❑

# PAIN / DISCOMFORT

I have no pain or discomfort ❑

I have moderate pain or discomfort ❑

I have extreme pain or discomfort ❑

# ANXIETY / DEPRESSION

I am not anxious or depressed ❑

I am moderately anxious or depressed ❑

I am extremely anxious or depressed ❑

*UK (English) © 2009 EuroQol Group EQ-5D™ is a trade mark of the EuroQol Group*

- We would like to know how good or bad your health is TODAY.

The best health you can imagine

- This scale is numbered from 0 to 100.
- 100 means the best health you can imagine. 0 means the worst health you can imagine.

100

### 95

90

- Mark an X on the scale to indicate how your health is TODAY. 85
- Now, please write the number you marked on the scale in the box below. 80

### 75

70

65

60

55

YOUR HEALTH TODAY =

50

45

40

35

30

25

20

15

10

5

0

The worst health you can imagine

*UK (English) © 2009 EuroQol Group EQ-5D™ is a trade mark of the EuroQol Group*

How severe is your pain *now*? Place a vertical mark on the line below to indicate how bad you feel your pain is *now*.

#### No pain Worst imaginable pain

Please indicate if you have taken any analgesics/pain medication in the *24 hours prior* to attending today:

Type of medication(s):

Time(s) at which medication were taken (eg. 8am):

Dose(s) of medication:

**POST-OP (after LA has worn off) DATE & TIME:**

Under each heading, please tick the ONE box that best describes your health TODAY.

# MOBILITY

I have no problems in walking about ❑

I have slight problems in walking about ❑

I have moderate problems in walking about ❑

I have severe problems in walking about ❑

I am unable to walk about ❑

# SELF-CARE

I have no problems washing or dressing myself ❑

I have slight problems washing or dressing myself ❑

I have moderate problems washing or dressing myself ❑

I have severe problems washing or dressing myself ❑

I am unable to wash or dress myself ❑ **USUAL ACTIVITIES** *(e.g. work, study, housework, family or leisure activities)*

I have no problems doing my usual activities ❑

I have slight problems doing my usual activities ❑

I have moderate problems doing my usual activities ❑

I have severe problems doing my usual activities ❑

I am unable to do my usual activities ❑

# PAIN / DISCOMFORT

I have no pain or discomfort ❑

I have slight pain or discomfort ❑

I have moderate pain or discomfort ❑

I have severe pain or discomfort ❑

I have extreme pain or discomfort ❑

# ANXIETY / DEPRESSION

I am not anxious or depressed ❑

I am slightly anxious or depressed ❑

I am moderately anxious or depressed ❑

I am severely anxious or depressed ❑

I am extremely anxious or depressed ❑

*UK (English) © 2009 EuroQol Group EQ-5D™ is a trade mark of the EuroQol Group*

**POST-OP (after LA has worn off) DATE & TIME:**

Under each heading, please tick the ONE box that best describes your health TODAY.

# MOBILITY

I have no problems in walking about ❑

I have some problems in walking about ❑

I am confined to bed ❑

# SELF-CARE

I have no problems washing or dressing myself ❑

I have some problems washing or dressing myself ❑

I am unable to wash or dress myself ❑ **USUAL ACTIVITIES** *(e.g. work, study, housework, family or leisure activities)*

I have no problems doing my usual activities ❑

I have some problems doing my usual activities ❑

I am unable to perform my usual activities ❑

# PAIN / DISCOMFORT

I have no pain or discomfort ❑

I have moderate pain or discomfort ❑

I have extreme pain or discomfort ❑

# ANXIETY / DEPRESSION

I am not anxious or depressed ❑

I am moderately anxious or depressed ❑

I am extremely anxious or depressed ❑

*UK (English) © 2009 EuroQol Group EQ-5D™ is a trade mark of the EuroQol Group*

- We would like to know how good or bad your health is TODAY.

The best health you can imagine

- This scale is numbered from 0 to 100.
- 100 means the best health you can imagine. 0 means the worst health you can imagine.

100

### 95

90

- Mark an X on the scale to indicate how your health is TODAY. 85
- Now, please write the number you marked on the scale in the box below. 80

### 75

70

65

60

55

YOUR HEALTH TODAY =

50

45

40

35

30

25

20

15

10

5

0

The worst health you can imagine

*UK (English) © 2009 EuroQol Group EQ-5D™ is a trade mark of the EuroQol Group*

How severe is your pain *now*? Place a vertical mark on the line below to indicate how bad you feel your pain is *now*.

#### No pain Worst imaginable pain

Please indicate if you have taken any analgesics/pain medication *after today’s dental visit*:

Type of medication(s):

Time(s) at which medication were taken (eg. 8am):

Dose(s) of medication:

**DAY 1 POST-OP DATE & TIME:**

Under each heading, please tick the ONE box that best describes your health TODAY.

# MOBILITY

I have no problems in walking about ❑

I have slight problems in walking about ❑

I have moderate problems in walking about ❑

I have severe problems in walking about ❑

I am unable to walk about ❑

# SELF-CARE

I have no problems washing or dressing myself ❑

I have slight problems washing or dressing myself ❑

I have moderate problems washing or dressing myself ❑

I have severe problems washing or dressing myself ❑

I am unable to wash or dress myself ❑ **USUAL ACTIVITIES** *(e.g. work, study, housework, family or leisure activities)*

I have no problems doing my usual activities ❑

I have slight problems doing my usual activities ❑

I have moderate problems doing my usual activities ❑

I have severe problems doing my usual activities ❑

I am unable to do my usual activities ❑

# PAIN / DISCOMFORT

I have no pain or discomfort ❑

I have slight pain or discomfort ❑

I have moderate pain or discomfort ❑

I have severe pain or discomfort ❑

I have extreme pain or discomfort ❑

# ANXIETY / DEPRESSION

I am not anxious or depressed ❑

I am slightly anxious or depressed ❑

I am moderately anxious or depressed ❑

I am severely anxious or depressed ❑

I am extremely anxious or depressed ❑

*UK (English) © 2009 EuroQol Group EQ-5D™ is a trade mark of the EuroQol Group*

**DAY 1 POST-OP DATE & TIME:**

Under each heading, please tick the ONE box that best describes your health TODAY.

# MOBILITY

I have no problems in walking about ❑

I have some problems in walking about ❑

I am confined to bed ❑

# SELF-CARE

I have no problems washing or dressing myself ❑

I have some problems washing or dressing myself ❑

I am unable to wash or dress myself ❑ **USUAL ACTIVITIES** *(e.g. work, study, housework, family or leisure activities)*

I have no problems doing my usual activities ❑

I have some problems doing my usual activities ❑

I am unable to perform my usual activities ❑

# PAIN / DISCOMFORT

I have no pain or discomfort ❑

I have moderate pain or discomfort ❑

I have extreme pain or discomfort ❑

# ANXIETY / DEPRESSION

I am not anxious or depressed ❑

I am moderately anxious or depressed ❑

I am extremely anxious or depressed ❑

*UK (English) © 2009 EuroQol Group EQ-5D™ is a trade mark of the EuroQol Group*

- We would like to know how good or bad your health is TODAY.

The best health you can imagine

- This scale is numbered from 0 to 100.
- 100 means the best health you can imagine. 0 means the worst health you can imagine.

100

### 95

90

- Mark an X on the scale to indicate how your health is TODAY. 85
- Now, please write the number you marked on the scale in the box below. 80

### 75

70

65

60

55

YOUR HEALTH TODAY =

50

45

40

35

30

25

20

15

10

5

0

The worst health you can imagine

*UK (English) © 2009 EuroQol Group EQ-5D™ is a trade mark of the EuroQol Group*

How severe is your pain *now*? Place a vertical mark on the line below to indicate how bad you feel your pain is *now*.

#### No pain Worst imaginable pain

Please indicate if you have taken any analgesics/pain medication in the *last 24 hours*:

Type of medication(s):

Time(s) at which medication were taken (eg. 8am):

Dose(s) of medication:

**DAY 3 POST-OP DATE & TIME:**

Under each heading, please tick the ONE box that best describes your health TODAY.

# MOBILITY

I have no problems in walking about ❑

I have slight problems in walking about ❑

I have moderate problems in walking about ❑

I have severe problems in walking about ❑

I am unable to walk about ❑

# SELF-CARE

I have no problems washing or dressing myself ❑

I have slight problems washing or dressing myself ❑

I have moderate problems washing or dressing myself ❑

I have severe problems washing or dressing myself ❑

I am unable to wash or dress myself ❑ **USUAL ACTIVITIES** *(e.g. work, study, housework, family or leisure activities)*

I have no problems doing my usual activities ❑

I have slight problems doing my usual activities ❑

I have moderate problems doing my usual activities ❑

I have severe problems doing my usual activities ❑

I am unable to do my usual activities ❑

# PAIN / DISCOMFORT

I have no pain or discomfort ❑

I have slight pain or discomfort ❑

I have moderate pain or discomfort ❑

I have severe pain or discomfort ❑

I have extreme pain or discomfort ❑

# ANXIETY / DEPRESSION

I am not anxious or depressed ❑

I am slightly anxious or depressed ❑

I am moderately anxious or depressed ❑

I am severely anxious or depressed ❑

I am extremely anxious or depressed ❑

*UK (English) © 2009 EuroQol Group EQ-5D™ is a trade mark of the EuroQol Group*

**DAY 3 POST-OP DATE & TIME:**

Under each heading, please tick the ONE box that best describes your health TODAY.

# MOBILITY

I have no problems in walking about ❑

I have some problems in walking about ❑

I am confined to bed ❑

# SELF-CARE

I have no problems washing or dressing myself ❑

I have some problems washing or dressing myself ❑

I am unable to wash or dress myself ❑ **USUAL ACTIVITIES** *(e.g. work, study, housework, family or leisure activities)*

I have no problems doing my usual activities ❑

I have some problems doing my usual activities ❑

I am unable to perform my usual activities ❑

# PAIN / DISCOMFORT

I have no pain or discomfort ❑

I have moderate pain or discomfort ❑

I have extreme pain or discomfort ❑

# ANXIETY / DEPRESSION

I am not anxious or depressed ❑

I am moderately anxious or depressed ❑

I am extremely anxious or depressed ❑

*UK (English) © 2009 EuroQol Group EQ-5D™ is a trade mark of the EuroQol Group*

- We would like to know how good or bad your health is TODAY.

The best health you can imagine

- This scale is numbered from 0 to 100.
- 100 means the best health you can imagine. 0 means the worst health you can imagine.

100

### 95

90

- Mark an X on the scale to indicate how your health is TODAY. 85
- Now, please write the number you marked on the scale in the box below. 80

### 75

70

65

60

55

YOUR HEALTH TODAY =

50

45

40

35

30

25

20

15

10

5

0

The worst health you can imagine

*UK (English) © 2009 EuroQol Group EQ-5D™ is a trade mark of the EuroQol Group*

How severe is your pain *now*? Place a vertical mark on the line below to indicate how bad you feel your pain is *now*.

#### No pain Worst imaginable pain

Please indicate if you have taken any analgesics/pain medication in the *last 48 hours*:

Type of medication(s):

Time(s) at which medication were taken (eg. 8am):

Dose(s) of medication:

**DAY 5 POST-OP DATE & TIME:**

Under each heading, please tick the ONE box that best describes your health TODAY.

# MOBILITY

I have no problems in walking about ❑

I have slight problems in walking about ❑

I have moderate problems in walking about ❑

I have severe problems in walking about ❑

I am unable to walk about ❑

# SELF-CARE

I have no problems washing or dressing myself ❑

I have slight problems washing or dressing myself ❑

I have moderate problems washing or dressing myself ❑

I have severe problems washing or dressing myself ❑

I am unable to wash or dress myself ❑ **USUAL ACTIVITIES** *(e.g. work, study, housework, family or leisure activities)*

I have no problems doing my usual activities ❑

I have slight problems doing my usual activities ❑

I have moderate problems doing my usual activities ❑

I have severe problems doing my usual activities ❑

I am unable to do my usual activities ❑

# PAIN / DISCOMFORT

I have no pain or discomfort ❑

I have slight pain or discomfort ❑

I have moderate pain or discomfort ❑

I have severe pain or discomfort ❑

I have extreme pain or discomfort ❑

# ANXIETY / DEPRESSION

I am not anxious or depressed ❑

I am slightly anxious or depressed ❑

I am moderately anxious or depressed ❑

I am severely anxious or depressed ❑

I am extremely anxious or depressed ❑

*UK (English) © 2009 EuroQol Group EQ-5D™ is a trade mark of the EuroQol Group*

**DAY 5 POST-OP DATE & TIME:**

Under each heading, please tick the ONE box that best describes your health TODAY.

# MOBILITY

I have no problems in walking about ❑

I have some problems in walking about ❑

I am confined to bed ❑

# SELF-CARE

I have no problems washing or dressing myself ❑

I have some problems washing or dressing myself ❑

I am unable to wash or dress myself ❑ **USUAL ACTIVITIES** *(e.g. work, study, housework, family or leisure activities)*

I have no problems doing my usual activities ❑

I have some problems doing my usual activities ❑

I am unable to perform my usual activities ❑

# PAIN / DISCOMFORT

I have no pain or discomfort ❑

I have moderate pain or discomfort ❑

I have extreme pain or discomfort ❑

# ANXIETY / DEPRESSION

I am not anxious or depressed ❑

I am moderately anxious or depressed ❑

I am extremely anxious or depressed ❑

*UK (English) © 2009 EuroQol Group EQ-5D™ is a trade mark of the EuroQol Group*

- We would like to know how good or bad your health is TODAY.

The best health you can imagine

- This scale is numbered from 0 to 100.
- 100 means the best health you can imagine. 0 means the worst health you can imagine.

100

### 95

90

- Mark an X on the scale to indicate how your health is TODAY. 85
- Now, please write the number you marked on the scale in the box below. 80

### 75

70

65

60

55

YOUR HEALTH TODAY =

50

45

40

35

30

25

20

15

10

5

0

The worst health you can imagine

*UK (English) © 2009 EuroQol Group EQ-5D™ is a trade mark of the EuroQol Group*

How severe is your pain *now*? Place a vertical mark on the line below to indicate how bad you feel your pain is *now*.

#### No pain Worst imaginable pain

Please indicate if you have taken any analgesics/pain medication in the *last 48 hours*:

Type of medication(s):

Time(s) at which medication were taken (eg. 8am):

Dose(s) of medication:

**DAY 7 POST-OP DATE & TIME:**

Under each heading, please tick the ONE box that best describes your health TODAY.

# MOBILITY

I have no problems in walking about ❑

I have slight problems in walking about ❑

I have moderate problems in walking about ❑

I have severe problems in walking about ❑

I am unable to walk about ❑

# SELF-CARE

I have no problems washing or dressing myself ❑

I have slight problems washing or dressing myself ❑

I have moderate problems washing or dressing myself ❑

I have severe problems washing or dressing myself ❑

I am unable to wash or dress myself ❑ **USUAL ACTIVITIES** *(e.g. work, study, housework, family or leisure activities)*

I have no problems doing my usual activities ❑

I have slight problems doing my usual activities ❑

I have moderate problems doing my usual activities ❑

I have severe problems doing my usual activities ❑

I am unable to do my usual activities ❑

# PAIN / DISCOMFORT

I have no pain or discomfort ❑

I have slight pain or discomfort ❑

I have moderate pain or discomfort ❑

I have severe pain or discomfort ❑

I have extreme pain or discomfort ❑

# ANXIETY / DEPRESSION

I am not anxious or depressed ❑

I am slightly anxious or depressed ❑

I am moderately anxious or depressed ❑

I am severely anxious or depressed ❑

I am extremely anxious or depressed ❑

*UK (English) © 2009 EuroQol Group EQ-5D™ is a trade mark of the EuroQol Group*

**DAY 7 POST-OP DATE & TIME:**

Under each heading, please tick the ONE box that best describes your health TODAY.

# MOBILITY

I have no problems in walking about ❑

I have some problems in walking about ❑

I am confined to bed ❑

# SELF-CARE

I have no problems washing or dressing myself ❑

I have some problems washing or dressing myself ❑

I am unable to wash or dress myself ❑ **USUAL ACTIVITIES** *(e.g. work, study, housework, family or leisure activities)*

I have no problems doing my usual activities ❑

I have some problems doing my usual activities ❑

I am unable to perform my usual activities ❑

# PAIN / DISCOMFORT

I have no pain or discomfort ❑

I have moderate pain or discomfort ❑

I have extreme pain or discomfort ❑

# ANXIETY / DEPRESSION

I am not anxious or depressed ❑

I am moderately anxious or depressed ❑

I am extremely anxious or depressed ❑

*UK (English) © 2009 EuroQol Group EQ-5D™ is a trade mark of the EuroQol Group*

- We would like to know how good or bad your health is TODAY.

The best health you can imagine

- This scale is numbered from 0 to 100.
- 100 means the best health you can imagine. 0 means the worst health you can imagine.

100

### 95

90

- Mark an X on the scale to indicate how your health is TODAY. 85
- Now, please write the number you marked on the scale in the box below. 80

### 75

70

65

60

55

YOUR HEALTH TODAY =

50

45

40

35

30

25

20

15

10

5

0

The worst health you can imagine

*UK (English) © 2009 EuroQol Group EQ-5D™ is a trade mark of the EuroQol Group*

How severe is your pain *now*? Place a vertical mark on the line below to indicate how bad you feel your pain is *now*.

#### No pain Worst imaginable pain

Please indicate if you have taken any analgesics/pain medication in the *last 48 hours*:

Type of medication(s):

Time(s) at which medication were taken (eg. 8am):

Dose(s) of medication:

***Oral Health-Related Quality of Life (OHIP – 14)***

|  | **Never** | **Occasionally** | **Fairly often** | **Very often** |
| --- | --- | --- | --- | --- |
| **1. Have you had trouble pronouncing any words because of problems with your teeth, mouth or dentures?** |  |  |  |  |
| **2. Have you felt that your sense of taste has worsened because of problems with your teeth, mouth or dentures?** |  |  |  |  |
| **3. Have you had painful aching in your mouth?** |  |  |  |  |
| **4. Have you found it uncomfortable to eat any foods because of problems with your teeth, mouth or dentures?** |  |  |  |  |
| **5. Have you been self conscious because of your teeth, mouth or dentures?** |  |  |  |  |
| **6. Have you felt tense because of problems with your teeth, mouth or dentures?** |  |  |  |  |
| **7. Has your diet been unsatisfactory because of problems with your teeth, mouth or dentures?** |  |  |  |  |
| **8. Have you had to interrupt meals because of problems with your teeth, mouth or dentures?** |  |  |  |  |
| **9. Have you found it difficult to relax because of problems with your teeth, mouth or dentures?** |  |  |  |  |
| **10. Have you been a bit embarrassed because of problems with your teeth, mouth or dentures?** |  |  |  |  |
| **11. Have you been a bit irritable with other people because of problems with your teeth, mouth or dentures?** |  |  |  |  |

| **12. Have you had difficulty doing your usual jobs because of problems with your teeth, mouth or dentures?** |  |  |  |  |
| --- | --- | --- | --- | --- |
| **13. Have you felt that life in general was less satisfying because of problems with your teeth, mouth or dentures?** |  |  |  |  |
| **14. Have you been totally unable to function because of problems with your teeth, mouth or dentures?** |  |  |  |  |
